# *Leishmania enriettii* (Muniz & Medina, 1948): A highly diverse parasite is here to stay

**DOI:** 10.1371/journal.ppat.1006303

**Published:** 2017-05-25

**Authors:** Larissa F. Paranaiba, Lucélia J. Pinheiro, Ana C. Torrecilhas, Diego H. Macedo, Armando Menezes-Neto, Wagner L. Tafuri, Rodrigo P. Soares

**Affiliations:** 1 Departamento de Parasitologia, Universidade Federal Minas Gerais (UFMG), Belo Horizonte, Minas Gerais, Brazil; 2 Departamento de Patologia, Universidade Federal Minas Gerais (UFMG), Belo Horizonte, Minas Gerais, Brazil; 3 Universidade Federal de São Paulo (UNIFESP), Diadema, São Paulo, Brazil; 4 Centro de Pesquisas René Rachou/Fundação Oswaldo Cruz (Fiocruz), Belo Horizonte, Minas Gerais, Brazil; University of Wisconsin Medical School, UNITED STATES

Leishmaniases are a spectrum of diseases caused by protozoans from the genus *Leishmania* (Kinetoplastida: Trypanosomatidae) and are divided into 2 main clinical forms: tegumentary leishmaniasis (TL) and visceral leishmaniasis (VL). Transmission occurs after the bite of sandfly vectors (Diptera: Phlebotominae) when females take a blood meal from the vertebrate host [1].

In the New World, several species of *Leishmania* (~20) cause disease to man, the symptoms and epidemiology of which vary depending on species. However, there are species that are nonpathogenic to humans, such as *L*. *enriettii*. In 1946, Medina observed ear lesions in 2 farm-reared guinea pigs (*Cavia porcellus* [Rodentia: Cavida]) from the neighboring state of São Paulo. After lesion analysis, *Leishmania* was confirmed as the pathogen. The complete *L*. *enriettii* description was published by Muniz and Medina in 1948 at the Federal University of Paraná, Brazil [2]. Although this species has been used as a model for cutaneous leishmaniasis (CL), many aspects of its biology remain unknown. In the past 6 years, an increased interest has emerged after the finding of a similar isolate in the red kangaroo (*Macrofus rufus*) in Australia [3]. This article aims to summarize some of the most important publications on this unique pathogen. It demonstrates a high phenotypic plasticity, being able to infect different vertebrate hosts and vectors. It also discusses recent human and veterinary infections due to other *L*. *enriettii* complex members.

## *L*. *enriettii*: Vertebrate hosts

**Fig 1:**
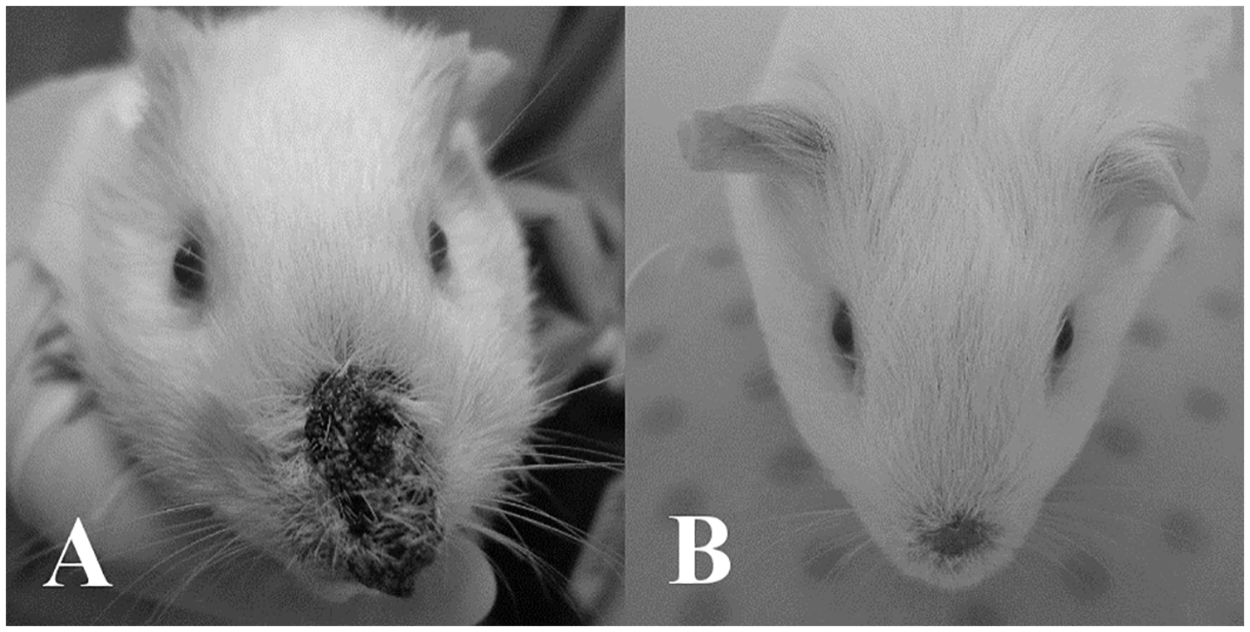
*Cavia porcellus* infected with *Leishmania enriettii* in the nose. (A) Four to 7 weeks of infection and (B) healed lesion after 8–10 weeks of infection.

After *L*. *enriettii* discovery in *C*. *porcellus* in the 1940s [4], the authors failed to infect monkeys, dogs, and wild guinea pigs (*C*. *aperea)*. They succeeded in infecting only 1 hamster out of 8 animals, and its lesion was poorly infected [2]. This is very likely to occur with wild reservoirs of *Leishmania*, such as opossums and armadillos, which, in nature, harbor low parasite densities without visible infection. At that time, no molecular approaches were available, opening the possibility of detecting *L*. *enriettii* in wild reservoirs other than *C*. *aperea* (Fig 1).

In spite of that, *L*. *enriettii* epidemiological studies involving hosts in Brazil are scarce, and new information on this parasite did not emerge until almost 50 years later. Two infected guinea pigs from the city of Capão Bonito (São Paulo state) and 3 from Campina Grande do Sul (Paraná state) were found. Species status was confirmed by isoenzyme analysis, with the description of a zymodeme polymorphism in the strains from Paraná [5]. Those data suggest that *L*. *enriettii* seems located in the Southern parts of Brazil, but its presence in other states should be prospected. A recent interesting study on *Leishmania* detection in road-killed wild animals in São Paulo found 1 *C*. *aperea* (1/4) infected with *Leishmania*. Although this study did not type the species, it provided some evidence that sylvatic *C*. *aperea* could harbor *Leishmania* [6]. Although the above-mentioned rodents have been considered the most common reservoirs for *L*. *enriettii* in Brazil, the finding of a putative similar isolate in captive red kangaroos (*M*. *rufus*), northern wallaroos (*Macropus robustus woodwardii*), black wallaroos (*M*. *bernardus*), and agile wallabies (*M*. *agilis*) was a landmark in the leishmaniasis epidemiology in Australia. The complete identification at species level only came in 2011 [3], classifying this isolate as a member of the *L*. *enriettii* complex [7].

## *L*. *enriettii*: Possible invertebrate vectors

Only 20 years after the description of *L*. *enriettii* (1967), evidence appeared regarding its invertebrate host [8]. A survey of phlebotomine fauna in Paraná state identified *Lutzomyia monticola* and *Lutzomyia correalimai* in the vicinities where the guinea pigs were infected. Experimental sandfly infections on those guinea pigs were performed where 60% (6/10) of *L*. *monticula* exhibited a very high infection in their midguts. For this reason, *L*. *enriettii* was considered a suprapylarian species and included in the subgenus *Leishmania*. The authors recovered those parasites from sandflies and inoculated naive guinea pigs. However, no development of infection was observed, and transmission through *L*. *monticola* bite has yet to be determined.

Since no *L*. *monticola* laboratory colony is available, transmission experiments are difficult to perform. For this reason, it is a challenge to ascertain its current status as a *L*. *enriettii* vector. This vector is widely spread from southern to northern Brazilian states. Although *L*. *monticola* has no human medical importance, it is very anthropophilic and is often captured together with vectors of CL and VL. More importantly, its distribution overlaps with domestic *C*. *porcellus* and wild *C*. *aperea*, reinforcing further studies with this vector.

In spite of having many sandfly species in Australia, the first evidence that midges from the subgenus *Forcipomyia* (*Lasiohelea*) (Diptera: Ceratopogonidae) could be an alternative vector of *Leishmania* has emerged. Although this vector fell into most of the Killick-Kendrick criteria, transmission still needs to be demonstrated after its colonization [3]. It is important to mention that this work triggered a recent increase in studying *L*. *enriettii* by many groups. Recently, 2 other ceratopogonids (*Culicoides nubeculosus* and *Culicoides sonorensis)* and *Lutzomyia longipalpis* were tested for *L*. *enriettii* infection using 2 strains (Brazilian and Australian) [9]. Only *C*. *sonorensis* was able to sustain infection, whereas *L*. *longipalpis* (permissive vector) developed moderate infections. Those data remarkably demonstrated *L*. *enriettii*’s ability to sustain and develop infection in different invertebrate hosts. Also, those studies stimulate further epidemiological investigations to identify other potential/alternative non–sandfly vectors.

## The *L*. *enriettii* complex members in human and veterinary infections

A detailed and historical review on *Leishmania* and sandflies was recently reported. However, a universal consensus regarding *Leishmania* classification is yet to be achieved [1], especially for the “*L*. *enrietti* complex.” In addition to *L*. *enriettii*, it may include *Leishmania martiniquensis* [10], “*Leishmania siamensis*” [11], and the Australian isolate [12]. In spite of the molecular techniques in establishing phylogenetic relationships among *Leishmania* species, a few studies have included *L*. *enriettii* complex members. The *L*. *martiniquensis* (strain MAR1) was isolated in Martinique Island, French Antilles, in 1995, causing diffuse CL in an HIV-infected patient [13]. A similar human isolate of *L*. *martiniquensis* causing VL was also reported from Thailand [14,15]. Finally, “*L*. *siamensis”* was first reported in Thailand as causing VL [16] and disseminated CL/VL in a patient with HIV. The isolate from this patient was named Trang strain and had 100% identity with *L*. *enrietti* after molecular analysis [11]. More recently, some isolates of *L*. *enriettii* complex members, probably “*L*. *siamensis*” and *L*. *martiniquensis*, were isolated from CL lesions in Ghana [15]. Detection of this species was also reported from a low number of cases in horses from Florida (2) [17] and Central Europe (6) [18] and in 1 cow from Switzerland [19]. However, as mentioned by Kwakye-Nuako et al. [15], although it appeared in the literature several times, the species “*L*. *siamensis*” was not formally described, and its name should be used in quotation marks.

The above-mentioned data suggest that members of *L*. *enriettii* complex are able to infect a wide range of different hosts, from rodents to humans, and to cause many clinical manifestations. In Table 1, we summarized some of the main isolates and reference strains of the *L*. *enriettii* complex members. Therefore, an international consortium for whole genome sequencing could be useful to ascertain the taxonomic status of this complex using those strains. This is of importance since the real status of “*L*. *siamensis”* and *L*. *martiniquensis* are still the subject of debate.

**Table 1:** Available isolates and strains of the *Leishmania enriettii* complex.

Species	World Health Organization code	Reference
*L*. *enriettii*	MCAV/BR/1945/L88 MCAV/BR/95/CUR3 MCAV/BR/1985/COBAIA_SP MCAV/BR/45/LV90 AM-2004	[20–23] [22] [23] [9] [12]
*“L*. *siamensis”*	Trang MHOM/GH/2012/GH5; LV757 MHOM/GH/2012/GH10; LV758 MHOM/GH/2012/GH11; LV759	[11] [15] [15] [15]
*L*. *martiniquensis*	MHOM/MQ/92/MAR 1 MHOM/MQ/92/MAR 2 MHOM/TH/2012/LSCM1	[10, 13, 24] [10] [14]

## Concluding remarks

Since its discovery in the 1940s, *L*. *enriettii* studies have been occurring in pulses. In the 1950s and 1960s, most of the studies were focused on its biology, transmission, and epidemiology. Later on, in the 1970s, some immunopathological and immunological studies appeared. In the 1990s, its use as a model for molecular biology was successfully employed. More recently, the findings of other putative *L*. *enriettii* complex members such as *L*. *martiniquensis* and “*L*. *siamensis”* have generated great interest in understanding the taxonomical relationships among those different isolates. Whole genome sequencing would be a very important tool to investigate such relationships and help to establish their real species status. The species of this complex exhibit a high phenotypic plasticity in being able to infect a wide range of vertebrate hosts, including humans and other vectors. They may also cause different symptoms, ranging from CL to VL. However, many aspects of their epidemiology are still unknown in the geographic areas where those parasites were isolated. Although there is strong evidence of some suspected vectors, another important gap yet to be demonstrated is vectorial transmission by either ceratopogonids or *L*. *monticula*.

In this context, almost 70 years after *L*. *enriettii* discovery, many questions and uncertainties about its biology, epidemiology, classification, and immunology remain unanswered. The finding of members of this complex on different continents and its veterinary and human medical importance, especially in patients with HIV, reinforces the need for more studies. Several groups in the world are now investigating those parasites, and an increase in the published papers in the past years has demonstrated that this parasite is here to stay.

## References

[1] Akhoundi M, Kuhls K, Cannet A, Votýpka J, Marty P. A Historical Overview of the Classification, Evolution, and Dispersion of *Leishmania* Parasites and Sandflies. PLoS Negl Trop Dis. 2016;10: 1–40. 10.1371/journal.pntd.0004349 PMC4777430 26937644

[2] Muniz J, Medina H. [Cutaneous leishmaniasis of the guinea pig, *Leishmania enriettii* n. sp]. Hospital (Rio J). 1948;33: 7–25. Portuguese. 18908199

[3] Dougall AM, Alexander B, Holt DC, Harris T, Sultan AH, Bates PA, et al Evidence incriminating midges (Diptera: Ceratopogonidae) as potential vectors of *Leishmania* in Australia. Int J Parasitol. Australian Society for Parasitology Inc.; 2011;41: 571–579. 10.1016/j.ijpara.2010.12.008 21251914

[4] Medina H. Estudos Sobre Leishmaniose: I. Primeiros Casos de Leishmaniose Espontânea Observados em Cobáios. Brazilian Arch Biol Technol. 2001;jubilee: 13–55.

[5] Thomaz-Soccol V, Pratlong F, Langue R, Castro E, Luz E, Dedet JP. New isolation of *Leishmania enriettii* Muniz and Medina, 1948 in Paranástate, Brazil, 50 years after the first description, and isoenzymatic polymorphism of the *L*. *enriettii* taxon. Ann Trop Med Parasitol. 1996;90: 491–5. 8915125 10.1080/00034983.1996.11813074

[6] Richini-Pereira VB, Marson PM, Hayasaka EY, Victoria C, da Silva RC, Langoni H. Molecular detection of *Leishmania* spp. in road-killed wild mammals in the Central Western area of the State of São Paulo, Brazil. J Venom Anim Toxins Incl Trop Dis. 2014;20: 27 10.1186/1678-9199-20-27 24963288 PMC4068874

[7] Dougall A, Shilton C, Low Choy J, Alexander B, Walton S. New reports of Australian cutaneous leishmaniasis in Northern Australian macropods. Epidemiol Infect. 2009;137: 1516–1520. 10.1017/S0950268809002313 19288959

[8] Luz E, Giovannoni M, Borba A. Infecção de *Lutzomyia monticola* por *Leishmania enriettii*. An da Fac Med da Univ Fed do Paraná. 1967;9: 121–128.

[9] Seblova V, Sadlova J, Vojtkova B, Votypka J, Carpenter S, Bates PA, et al The Biting Midge *Culicoides sonorensis* (Diptera: Ceratopogonidae) is Capable of Developing Late Stage Infections of *Leishmania enriettii*. PLoS Negl Trop Dis. 2015;9. 10.1371/journal.pntd.0004060 PMC4569557 26367424

[10] Desbois N, Pratlong F, Quist D, Dedet J-P. *Leishmania* (*Leishmania*) *martiniquensis* n. sp. (Kinetoplastida: Trypanosomatidae), description of the parasite responsible for cutaneous leishmaniasis in Martinique Island (French West Indies). Parasite. 2014;21: 1–4. 24626346 10.1051/parasite/2014011 PMC3952653

[11] Bualert L, Charungkiattikul W, Thongsuksai P, Mungthin M, Siripattanapipong S, Khositnithikul R, et al Case report: Autochthonous disseminated dermal and visceral leishmaniasis in an AIDS patient, Southern Thailand, caused by *Leishmania siamensis*. Am J Trop Med Hyg. 2012;86: 821–824. 22556080 10.4269/ajtmh.2012.11-0707 PMC3335686

[12] Rose K, Curtis J, Baldwin T, Mathis A, Kumar B, Sakthianandeswaren A, et al Cutaneous leishmaniasis in red kangaroos: Isolation and characterisation of the causative organisms. Int J Parasitol. 2004;34: 655–664. 10.1016/j.ijpara.2004.03.001 15111087

[13] Dedet JP, Roche B, Pratlong F, Caies-Quist D, Jouannelle J, Benichou JC, et al Diffuse cutaneous infection caused by a presumed monoxenous trypanosomatid in a patient infected with HIV. Trans R Soc Trop Med Hyg. 1995;89: 644–646. 8594682 10.1016/0035-9203(95)90427-1

[14] Pothirat T, Tantiworawit A, Chaiwarith R, Jariyapan N, Wannasan A, Siriyasatien P, et al First Isolation of *Leishmania* from Northern Thailand: Case Report, Identification as *Leishmania martiniquensis* and Phylogenetic Position within the *Leishmania enriettii* Complex. PLoS Negl Trop Dis. 2014;8: 1–8. 10.1371/journal.pntd.0003339 PMC4256172 25474647

[15] Kwakye-Nuako G, Mosore MT, Duplessis C, Bates MD, Puplampu N, Mensah-Attipoe I, et al First isolation of a new species of *Leishmania* responsible for human cutaneous leishmaniasis in Ghana and classification in the *Leishmania enriettii* complex. Int J Parasitol. Australian Society for Parasitology Inc.; 2015;45: 679–684. 10.1016/j.ijpara.2015.05.001 26099650

[16] Sukmee T, Siripattanapipong S, Mungthin M, Worapong J, Rangsin R, Samung Y, et al A suspected new species of *Leishmania*, the causative agent of visceral leishmaniasis in a Thai patient. Int J Parasitol. 2008;38: 617–622. 10.1016/j.ijpara.2007.12.003 18262531

[17] Reuss SM, Dunbar MD, Calderwood Mays MB, Owen JL, Mallicote MF, Archer LL, et al Autochthonous *Leishmania siamensis* in horse, Florida, USA. Emerg Infect Dis. 2012;18: 1545–1547. 10.3201/eid1809.120184 22932732 PMC3437729

[18] Muller N, Welle M, Lobsiger L, Stoffel MH, Boghenbor KK, Hilbe M, et al Occurrence of *Leishmania* sp. in cutaneous lesions of horses in Central Europe. Vet Parasitol. 2009;166: 346–351. 10.1016/j.vetpar.2009.09.001 19800739

[19] Lobsiger L, Muller N, Schweizer T, Frey CF, Wiederkehr D, Zumkehr B, et al An autochthonous case of cutaneous bovine leishmaniasis in Switzerland. Vet Parasitol. 2010;169: 408–414. 10.1016/j.vetpar.2010.01.022 20153118

[20] Thomaz-Soccol V, Lanotte G, Rioux JA, Pratlong F, Martini-Dumas A, Serres E. Phylogenetic taxonomy of New World *Leishmania*. Ann Parasitol Hum Comp. 1993;68: 104–106. 7692803

[21] Asato Y, Oshiro M, Myint CK, Yamamoto Y, Kato H, Marco JD, et al Phylogenic analysis of the genus *Leishmania* by cytochrome b gene sequencing. Exp Parasitol. Elsevier Inc.; 2009;121: 352–361. 10.1016/j.exppara.2008.12.013 19159626

[22] Noyes H, Pratlong F, Chance M, Ellis J, Lanotte G, Dedet JP. A previously unclassified trypanosomatid responsible for human cutaneous lesions in Martinique (French West Indies) is the most divergent member of the genus *Leishmania*. Parasitology. 2002;124: 17–24. 11811799 10.1017/s0031182001008927

[23] Paranaíba LF, de Assis RR, Nogueira PM, Torrecilhas AC, Campos JH, de O Silveira AC, et al *Leishmania enriettii*: biochemical characterisation of lipophosphoglycans (LPGs) and glycoinositolphospholipids (GIPLs) and infectivity to *Cavia porcellus*. Parasit Vectors. 2015;8: 31 10.1186/s13071-015-0633-8 25595203 PMC4311450

[24] Liautaud B, Vignier N, Miossec C, Plumelle Y, Kone M, Delta D, et al First case of visceral leishmaniasis caused by *Leishmania martiniquensis*. Am J Trop Med Hyg. 2015;92: 317–319. 10.4269/ajtmh.14-0205 25404076 PMC4347334

